# Transcriptional signatures of aberrant brain functional network topology in chronic low back pain with lumbar disc herniation

**DOI:** 10.3389/fnmol.2026.1790428

**Published:** 2026-08-12

**Authors:** Chuanxu Luo, Wanting Liu, Xiaoyi Liu, Han Xu, Hao Gu, Yapan Zhang, Fengchao Shi, Wenhui Li, Siyu Gu

**Affiliations:** 1 Department of Radiology, Yancheng Third People’s Hospital, Affiliated Hospital 6 of Nantong University, Yancheng, China; 2 Department of Orthopedics, Yancheng Third People’s Hospital, Affiliated Hospital 6 of Nantong University, Yancheng, China; 3 Yancheng Maternal and Child Health Care Hospital Affiliated to Yangzhou University, Yancheng, China

**Keywords:** brain network topology, chronic low back pain, graph theory, imaging transcriptomics, resting-state fMRI

## Abstract

**Objective:**

The central pathophysiology of chronic low back pain (CLBP) secondary to lumbar disc herniation (LDH) remains poorly understood, particularly the link between macroscale brain network dysfunction and its microscale biological underpinnings. This study aimed to bridge this critical gap by integrating resting-state fMRI, graph theory, and imaging transcriptomics.

**Methods:**

Resting-state fMRI data were acquired from 62 CLBP patients with LDH and 67 matched healthy controls (HCs). Whole-brain functional networks were constructed for each participant by calculating functional connectivity (FC) between 90 anatomical regions. Graph theoretical analysis was employed to derive global and nodal topological metrics. Group differences were identified and correlated with clinical variables. Crucially, these neuroimaging phenotypes were spatially mapped against the Allen Human Brain Atlas (AHBA) to uncover potential spatial molecular vulnerabilities.

**Results:**

At the connectional level, CLBP patients with LDH exhibited widespread hypoconnectivity across sensorimotor, ventral attention, and default mode networks. Further topological analysis revealed a profound focal degradation of hub function in the cingulate cortex, characterized by significantly reduced nodal degree, efficiency, and betweenness centrality (*p* < 0.001), alongside decreased nodal efficiency in the right supplementary motor area, while global network architecture was preserved. Critically, these deficits in cingulate hub topology were significantly correlated with pain intensity. Imaging transcriptomics linked this focal topological failure to genes enriched for neuroinflammatory and synaptic plasticity pathways.

**Conclusion:**

These findings reveal multi-level functional network alterations in CLBP with LDH, linking focal hub failure to potential spatial molecular vulnerabilities associated with neuroinflammation and synaptic remodeling.

## Introduction

Chronic low back pain (CLBP) is recognized as a leading cause of disability worldwide, imposing a substantial socioeconomic burden that is expected to increase by 2050 (GBD 2021 Low Back Pain Collaborators, 1990). Among various etiologies, CLBP caused by lumbar disc herniation (LDH) is particularly prevalent, characterized by an increasing incidence and a trend toward younger onset (Pojskic et al., 2024). CLBP not only impairs physical function but also diminishes quality of life, imposing a significant economic burden (Gatchel et al., 2007). Despite this, conventional treatments, such as pharmacotherapy or peripheral surgical interventions, often yield limited effectiveness (Chou et al., 2007). Current research increasingly looks beyond local tissue injury to the central nervous system (CNS), adopting a biopsychosocial perspective that accounts for the complex persistence of pain. Processes such as central sensitization, altered descending pain modulation, and maladaptive neuroplasticity are now considered key factors in the transition from acute to chronic pain and its maintenance in various CLBP subtypes, including LDH-related pain (Nijs et al., 2014; Martucci et al., 2014). This shift underscores the need for advanced neuroimaging to investigate the CNS mechanisms underlying chronic pain.

Resting-state functional magnetic resonance imaging (rs-fMRI) is a powerful non-invasive technique for exploring central mechanisms. Many studies have reported altered functional connectivity (FC) in CLBP, particularly within and between core networks such as the default mode network (DMN), ventral attention network (VAN), executive control network (ECN), and sensorimotor network (SMN) (Čeko et al., 2015; Mao et al., 2022; Zhang et al., 2024; Zu et al., 2024; Baumbach et al., 2022; Mao et al., 2023; Yang et al., 2023). These results suggest that CLBP is linked to widespread brain network remodeling, including dysregulation in circuits involved in pain perception, emotional regulation, and cognitive control (Chou et al., 2007; Nijs et al., 2014). However, pairwise connectivity analyses do not fully capture the complexity of brain organization, as the brain functions as an interconnected network. Graph theory analysis, by representing brain regions as nodes and connections as edges, allows for the quantitative assessment of global and local network properties, including clustering coefficient (local segregation), characteristic path length and network efficiency (global integration), and small-worldness (balance of local and global processing) (Mao et al., 2022; Selvaggi et al., 2021; Sporns, 2011). This approach enables a systems-level understanding of CLBP-related brain changes (Zhang et al., 2023). Prior work has used graph theory to identify reduced small-world properties, diminished global and local efficiency, and abnormal nodal metrics in regions such as the DMN and SN, and altered modular organization (Chou et al., 2007; Mao et al., 2022; Yang et al., 2023), indicating that CLBP may represent a “network disorder” involving widespread topological disruption. Therefore, a systematic investigation of whole-brain network topology is crucial for clarifying disease effects and identifying stable neuroimaging markers.

Nevertheless, mapping network topology alone does not reveal the biological basis of these changes. A key question remains: what molecular and cellular factors contribute to macro-scale network changes? Imaging transcriptomics provides a novel approach, integrating neuroimaging with spatial gene expression data (Selvaggi et al., 2021). The Allen Human Brain Atlas (AHBA) offers detailed maps of expression for over 20,000 genes (Hawrylycz et al., 2012). By spatially correlating a neuroimaging-derived map of pathology with genome-wide expression patterns, imaging transcriptomics can generate data-driven, testable hypotheses about the specific genes, biological pathways (e.g., neuroinflammation, synaptic plasticity), and cell types (e.g., microglia, astrocytes) that may underlie brain vulnerability and pathological remodeling in disease (Fornito et al., 2019; Guo et al., 2023).

Therefore, the present study aimed to integrate advanced network neuroscience and imaging transcriptomics to provide a multi-scale account of brain network dysfunction in CLBP patients with LDH. First, we calculated the FC of the whole brain. Subsequently, we utilized graph theoretical analysis of rs-fMRI data to comprehensively characterize the aberrant functional network topology in a cross-sectional sample of CLBP patients compared to healthy controls (HCs). Crucially, we then sought to identify the specific transcriptional signatures by correlating our neuroimaging findings with the AHBA.

## Methods

### Participants

A total of 62 CLBP patients with LDH and 67 age- and sex-matched HCs were recruited from the Affiliated Hospital 6 of Nantong University between January 2021 and October 2025. It should be noted that a subset of this cross-sectional sample (38 CLBP patients and 38 HCs) was utilized in our previous work exploring the temporal variance of functional edges (i.e., static and dynamic functional connectivity) (Gu et al., 2025). The current study addresses a fundamentally distinct scientific dimension: utilizing whole-brain graph theory to characterize macroscopic topological organization (nodal hubs) and, crucially, integrating these network alterations with the Allen Human Brain Atlas to uncover underlying spatial transcriptomic profiles. The full sample was retained here to ensure the necessary statistical power required for reliable spatial gene expression correlations. The study complied with the Declaration of Helsinki and received approval from the local Ethics Committee of the Affiliated Hospital 6 of Nantong University (Approval No. 2020–79). Written informed consent was obtained from all participants prior to enrolment.

All participants underwent a rigorous screening process. For inclusion, patients with CLBP were required to have: (1) clinically and radiologically confirmed lumbar disc herniation; (2) an age between 18 and 75 years; (3) pain persisting for at least 3 months; (4) a Visual Analogue Scale (VAS) score of 3 or higher; and (5) right-handedness. (6) regular use of analgesic medication, with details provided in Supplementary Table S1. At the time of clinical assessment and fMRI data acquisition, all enrolled patients were hospitalized presurgical candidates scheduled for lumbar decompression surgery. This stringent presurgical context naturally minimized clinical heterogeneity. Specifically, they shared a uniform prior treatment history characterized by the definitive failure of long-term conservative management, resulting in a severe, chronic-refractory pain state. To minimize extreme clinical variability, our group exhibited a relatively consistent clinical phenotype, which is detailed in Supplementary Table S1. Inclusion criteria for HCs were: (1) aged between 18 and 75 years; (2) no current pain; (3) no history of chronic pain; (4) absence of neurological or psychiatric disorders, diabetes, hypertension, hyperlipidemia, long-term smoking, or alcohol abuse history; and (5) right-handed. Participants were excluded if they were unable to comply with MRI scanning protocols, had contraindications for MRI, or exhibited brain lesions during routine scans.

### Clinical and neuropsychological assessment

Prior to the MRI scan, all participants underwent a comprehensive assessment. To control for potential pharmacological confounds, all patients were required to undergo a washout period, discontinuing analgesic medications for at least 24 h prior to the MRI acquisition. Patients dependent on opioids or psychotropic drugs were excluded from the study. For CLBP patients with LDH, this included a detailed medical history and evaluation of pain duration. To quantify pain intensity and its impact on daily life, patients completed the VAS and the Oswestry Disability Index (ODI) questionnaire (Huskisson, 1974; Fairbanks et al., 1980). The VAS required patients to rate their current pain intensity on a 10 cm scale, where higher scores reflect greater pain. The ODI is a 10-item survey assessing the level of functional disability across various activities, such as sitting, walking, and social life. The total ODI score is expressed as a percentage, with higher values indicating more severe functional impairment.

To evaluate emotional states, all participants completed the Self-Rating Anxiety Scale (SAS) and the Self-Rating Depression Scale (SDS) (Zung, 1965; Zung, 1971). Each of these validated 20-item questionnaires assesses the severity of anxiety and depressive symptoms. Raw scores are converted to a standard score, with a standard score of 50 or above indicating the presence of clinically relevant anxiety or depression. VAS and ODI were not administered to HCs.

### MRI data acquisition

All MRI data were acquired at the Affiliated Hospital 6 of Nantong University using a 3.0 Tesla GE Discovery 750 W scanner equipped with a 24-channel head coil. On the day of the scan, participants were instructed to refrain from consuming beverages containing alcohol or caffeine. During the procedure, foam padding and earplugs were used to minimize head motion and reduce scanner noise. Participants were instructed to remain calm and awake, lie still with their eyes closed, and avoid systematic thinking throughout the scan.

Rs-fMRI data were acquired using a gradient-echo echo-planar imaging (GRE-EPI) sequence. The scanning parameters were as follows: repetition time (TR) = 3,000 ms; echo time (TE) = 35 ms; flip angle (FA) = 90°; field of view (FOV) = 240 × 240 mm^2^; matrix size = 64 × 64; and slice thickness = 5 mm. A total of 128 functional volumes were collected for each participant, with a voxel size of 3.75 × 3.75 × 4 mm. Axial slices were acquired parallel to the anterior commissure-posterior commissure (AC-PC) line to ensure whole-brain coverage.

### Data preprocessing

Functional MRI data preprocessing was performed using the rs-fMRI Data Analysis Toolkit Plus (RESTplus v1.28; http://restfmri.net/forum/RESTplus) (Jia et al., 2019), a toolbox that operates within MATLAB (R2020b) and is based on functions from Statistical Parametric Mapping (SPM12; https://www.fil.ion.ucl.ac.uk/spm) (Ashburner and Friston, 2005).

The preprocessing pipeline began with the conversion of raw DICOM images to the NIFTI format. The first 10 time points of each functional run were discarded to ensure scanner signal stabilization, leaving the remaining 118 volumes for analysis. The subsequent preprocessing steps included: (1) Slice-timing correction, which adjusted for acquisition time differences between slices by referencing them to the middle slice. (2) Head motion correction, where images were realigned to the mean image. Participants with head motion exceeding 2 mm in translation or 2° in rotation were excluded from the study. Based on these criteria, 3 patients and 3 HCs were excluded. Furthermore, to verify the mitigation of residual micro-movements, the mean framewise displacement (FD) was calculated. An independent two-sample t-test revealed no significant difference in mean FD between the CLBP group (0.139 ± 0.108 mm) and the HC group (0.129 ± 0.090 mm) (*p* = 0.566 > 0.05). (3) Spatial normalization, where the realigned functional images were normalized to the standard Montreal Neurological Institute (MNI) space and resampled to an isotropic voxel size of 3 × 3 × 3 mm^3^. (4) Nuisance covariate regression, which involved removing signals of no interest using a linear regression model. These covariates included 24 head motion parameters (derived from the Friston-24 model), the mean signals from segmented white matter and cerebrospinal fluid, and any linear trends (linear detrending). (5) Temporal filtering, where a band-pass filter (0.01–0.1 Hz) was applied to the time series to isolate the low-frequency fluctuations characteristic of resting-state neural activity.

### Whole-brain functional network construction

The construction and analysis of the whole-brain functional networks were performed using the Graph Theoretical Network Analysis (GRETNA) toolbox (Wang et al., 2015). First, network nodes were defined by parcellating the preprocessed fMRI data into 90 regions of interest (ROIs) based on the Automated Anatomical Labeling 90 (AAL-90) atlas, excluding the cerebellum. The mean time series was then extracted for each of these ROIs.

Next, network edges were defined by calculating the Pearson correlation coefficient between the time series of every pair of ROIs, which generated a 90 × 90 correlation matrix for each participant. To improve normality for subsequent statistical analyses, these correlation coefficients were converted to *z*-scores using Fisher’s *r*-to-*z* transformation. The resulting 90 × 90 matrices, representing the weighted and undirected functional connectivity between all pairs of nodes, were used for all subsequent graph theoretical analyses.

### Graph theoretical analysis

To characterize the topological organization of the functional brain networks, we performed graph theoretical analysis directly on each participant’s weighted connectivity matrix using the GRETNA toolbox. This analysis computed a comprehensive set of metrics at both the global and nodal levels. The global metrics, assessing overall network integration and segregation, included the clustering coefficient (C*_p_*), characteristic path length (L*_p_*), normalized clustering coefficient (*γ*), normalized characteristic path length (*λ*), small-worldness (*σ*), global efficiency (E*_g_*), and local efficiency (E*_loc_*). Concurrently, nodal metrics were calculated to evaluate the importance of individual brain regions, which included nodal degree, nodal efficiency, and nodal betweenness centrality.

Acknowledging the sensitivity of topological properties to network density, we evaluated network topology across a broad sparsity range. This involved constructing networks from 0.05 to 0.40 with a step size of 0.01, a range selected to guarantee small-worldness ( σ ) > 1.1 and allow for a comprehensive assessment (Wang et al., 2015; Zhang et al., 2011; Keown et al., 2017). To mitigate bias stemming from selecting a single threshold, the area under the curve (AUC) for each topological coefficient over these sparsity thresholds was calculated, providing a summarized scalar of network topological characteristics.

### Gene expression data preprocessing

To create a regional gene expression map, we processed transcriptomic data from the six post-mortem donors in the AHBA using the abagen toolbox (Ross et al., 2021). The preprocessing pipeline involved several steps: (1) re-annotating microarray probes to their corresponding genes using updated genetic information; (2) selecting a single representative probe for each gene based on signal consistency; (3) applying a differential stability filter (stability > 0.1) across donors, which retained a final set of 15,634 reliably expressed genes from the initial pool; and (4) normalizing expression values within each donor before aggregating them to generate a single expression value for each gene within each of the 45 left-hemisphere regions of interest (ROIs) from the AAL-90 atlas, as data for the right hemisphere are available for only two of the six donors. This process resulted in a final 45 (ROIs) × 15,634 (genes) gene expression matrix that was used for all subsequent transcriptomic analyses.

### Imaging-transcriptomics association analysis

To identify the molecular substrates of the observed topological alterations, we performed a series of spatial association analyses. For each node attributes that demonstrated significant group differences, a corresponding spatial map of *t*-statistics was constructed across the 45 left-hemisphere ROIs. A separate Partial Least Squares (PLS) regression was then performed to identify a multivariate gene expression pattern that spatially covaried with each respective *t*-statistic map, using the 15,634-gene expression matrix as the independent variable. The statistical significance of each resulting PLS model was rigorously assessed using a spatial permutation test involving 5,000 permutations via the BrainSMASH toolbox to correct for spatial autocorrelation, as well as a standard permutation test of 5,000 permutations on the correlation between the PLS scores and the phenotype map. The contribution of individual genes to each significant PLS component was quantified using a bootstrap analysis with 5,000 resamples, with genes considered reliable contributors if their bootstrap ratio (*z*-score) exceeded |1.96|. Finally, for each analysis that yielded a significant association, the full list of genes, ranked by their *z*-scores, was submitted to the Metascape web portal^1^ for gene set enrichment analysis, specifically examining over-representation in Gene Ontology (GO) and Kyoto Encyclopedia of Genes and Genomes (KEGG) pathways.

### Statistical analysis

All statistical analyses were performed using SPSS software (Version 27.0). First, the normality of distribution for demographic and clinical variables was assessed. For normally distributed data, presented as mean ± standard deviation (SD), group differences were evaluated using independent two-sample *t*-tests. For data not following a normal distribution, presented as median and interquartile range (IQR), the non-parametric Mann–Whitney *U* test was used. The chi-square (*χ*^2^) test was used to assess group differences in sex distribution. A *p*-value < 0.05 was considered statistically significant for these initial analyses.

In addition to network-level topological analyses, we also examined group differences in the strength of individual functional connections. A General Linear Model (GLM) was performed for each of the 4,005 unique connections, with group as the main factor while controlling for age, sex, SAS scores and SDS scores as covariates. To account for the large number of comparisons, a stringent Bonferroni correction was applied to the resulting *p*-values. Connections with a corrected *p*-value < 0.05 were considered to show statistically significant group differences.

To identify group differences in brain network topology, we performed analyses on the derived graph theoretical metrics. Group comparisons for both global and nodal metrics were conducted using a GLM, with group as the main factor, while controlling for age, sex SAS scores and SDS scores as covariates. For the group comparisons of the summarized scalar (AUC) of the seven global network metrics, the False Discovery Rate (FDR) correction was applied to control for multiple comparisons across the 7 metrics, with an FDR-corrected *p*-value < 0.05 considered statistically significant. To account for multiple comparisons across the 90 nodes, a stringent Bonferroni correction was applied to the nodal analyses, and a corrected p -value < 0.05 was considered statistically significant.

Finally, to investigate the clinical relevance of the identified network alterations, partial correlation analyses were conducted within the CLBP patient group. We examined the relationship between the brain network metrics that showed significant group differences and the patients’ clinical characteristics (VAS scores, ODI scores, and pain duration). Crucially, to exclude the potential confounding effect of emotional distress, these analyses were performed controlling for age, sex, SAS scores, and SDS scores as covariates.

## Results

### Demographic and clinical characteristics

A total of 62 CLBP patients with LDH and 67 HCs were included in this study. The demographic and clinical characteristics of all participants are summarized in Table 1. There were no significant group differences in age (*p* = 0.697) or sex distribution (*p* = 0.465). However, patients with CLBP reported significantly higher scores on both the SAS (*p* < 0.001) and the SDS (*p* < 0.001) compared to the HCs. Among the CLBP group, 45 patients (72.6%) were managed with NSAIDs (e.g., Celecoxib, Ibuprofen), while 10 patients (16.1%) used gabapentinoids. Seven patients reported intermittent use of over-the-counter analgesics.

**Table 1:** The demographical information and clinical characteristics of patients with CLBP and HCs.

Characteristics	CLBP	HCs	*t/z*/*χ*^2^	*p* value
*N*	62	67	–	–
Age (year)	52.50 (44.75–62.25)	53.00 (39.00–61.00)	−0.389	0.697
Gender (Male/Female)	28/34	26/41	0.534	0.465
VAS	7.60 (7.17–8.23)	–	–	–
ODI	64.00 (56.75–68.00)	–	–	–
Pain duration (year)	3.50 (1.00–8.00)	–	–	–
SAS	55.23 ± 8.93	31.37 ± 7.97	15.834	<0.001
SDS	58.00 (48.00–62.00)	31.00 (27.00–37.00)	−8.891	<0.001

Data are presented as mean ± standard deviation for normally distributed variables, median (interquartile range) for non-normally distributed variables, or n for categorical data. Group differences were assessed using independent two-sample *t*-tests or Mann–Whitney U tests for continuous variables, and the Pearson’s chi-square (*χ*^2^) test for categorical variables. A *p*-value < 0.05 was considered statistically significant.

CLBP, chronic low back pain; HCs, healthy controls; VAS, Visual Analogue Scale; ODI, Oswestry Disability Index; SAS, Self-Rating Anxiety Scale; SDS, Self-Rating Depression Scale.

### Altered FC between groups

**Figure 1:**
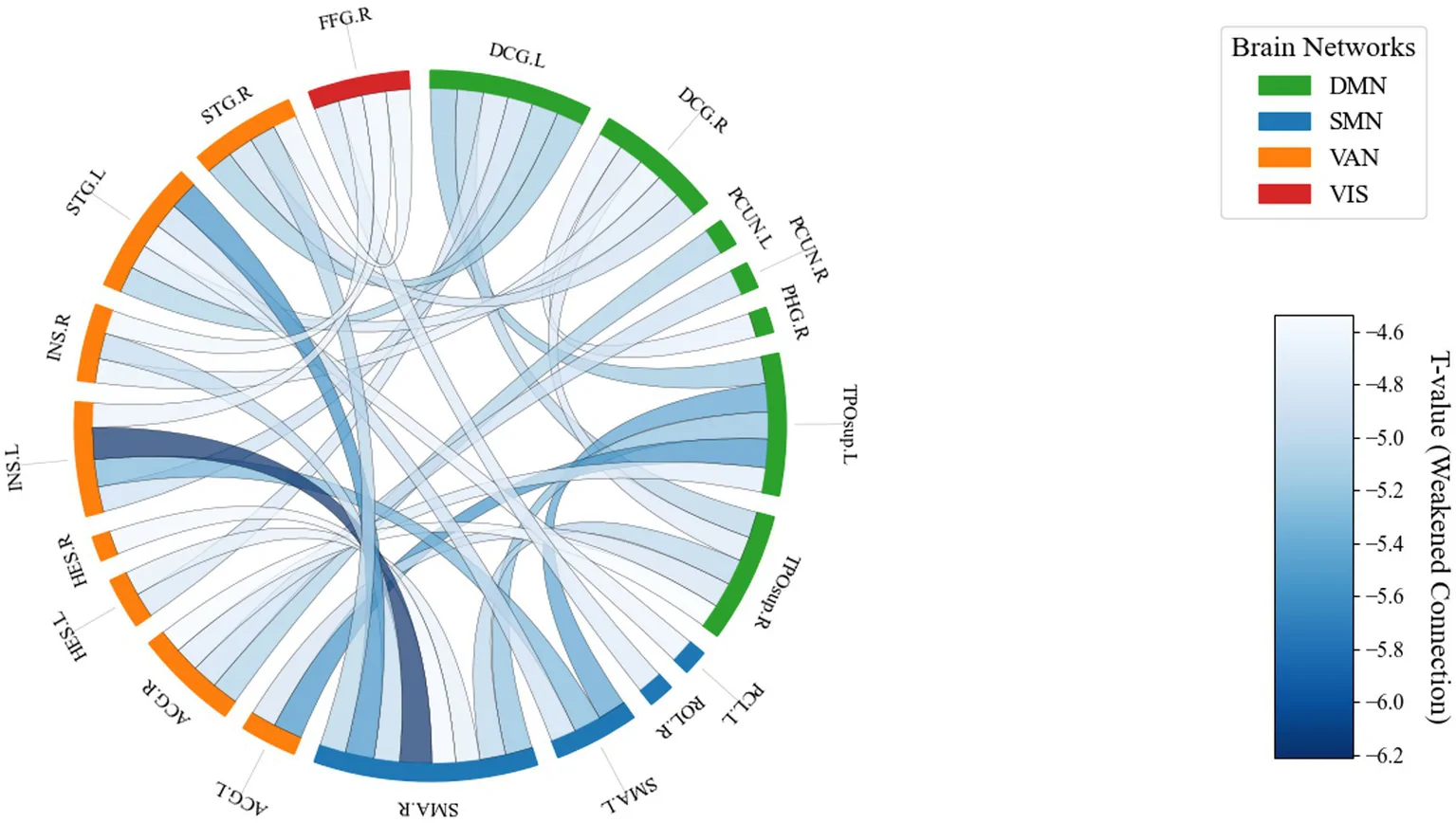
Widespread functional hypoconnectivity in CLBP patients with LDH. All results survived the stringent Bonferroni correction for multiple comparisons (*p* < 0.05, corrected). CLBP, Chronic Low Back Pain; FC, Functional Connectivity; ACG, Anterior Cingulate Gyrus; DCG, Dorsal Cingulate Gyrus; FFG, Fusiform Gyrus; HES, Heschl’s Gyrus; INS, Insula; PCL, Paracentral Lobule; PCUN, Precuneus; PHG, Parahippocampal Gyrus; ROL, Rolandic Operculum; SMA, Supplementary Motor Area; STG, Superior Temporal Gyrus; TPOsup, Temporal Pole, Superior Part; L, left; R, right; SMN, Somatomotor Network; VAN, Ventral Attention Network; DMN, Default Mode Network; VIS, Visual Network.

Whole-brain analysis revealed widespread reduced FC in CLBP patients with LDH compared to HCs (*p* < 0.05, Bonferroni corrected), primarily involving FC within and between the SMN, VAN, DMN, and visual network (VIS). Specifically, key nodes of the SMN (e.g., bilateral SMA, left PCL) exhibited significantly reduced connectivity with core hubs of the VAN (e.g., bilateral INS, STG, HES). Furthermore, impaired information exchange was prominent between the VAN and DMN, evidenced by reduced connectivity between VAN hubs (bilateral ACG, INS) and DMN regions (bilateral DCG, PCUN, TPOsup). This pattern of hypoconnectivity also extended to connections within the DMN itself (e.g., DCG to PHG and TPOsup), and was observed in cross-modal pathways, with weakened connectivity between the Visual Network (FFG) and nodes of both the VAN (INS, STG) and the SMN (ROL). All detailed results are presented in Table 2 and visualized in Figure 1.

**Table 2:** Significant reductions in functional connectivity in CLBP patients with LDH.

Brain region 1	Network 1	Brain region 2	Network 2	*p* value	*t* value
SMA.L	SMN	INS.L	VAN	< 0.001	−5.32
SMA.R	SMN	INS.L	VAN	< 0.001	−6.21
SMA.R	SMN	INS.R	VAN	< 0.001	−4.99
INS.L	VAN	DCG.L	DMN	< 0.001	−4.90
INS.R	VAN	DCG.R	DMN	< 0.001	−4.67
DCG.R	DMN	PHG.R	DMN	< 0.001	−4.70
ROL.R	SMN	FFG.R	VIS	< 0.001	−4.78
INS.L	VAN	FFG.R	VIS	< 0.001	−4.66
INS.R	VAN	FFG.R	VIS	< 0.001	−4.61
ACG.R	VAN	PCUN.L	DMN	< 0.001	−5.09
ACG.R	VAN	PCUN.R	DMN	< 0.001	−4.91
SMA.R	SMN	HES.L	VAN	< 0.001	−4.69
DCG.L	DMN	HES.L	VAN	< 0.001	−4.82
SMA.R	SMN	HES.R	VAN	< 0.001	−4.59
SMA.L	SMN	STG.L	VAN	< 0.001	−4.90
SMA.R	SMN	STG.L	VAN	< 0.001	−5.59
DCG.L	DMN	STG.L	VAN	< 0.001	−5.10
DCG.R	DMN	STG.L	VAN	< 0.001	−4.78
PCL.L	SMN	STG.L	VAN	< 0.001	−4.66
SMA.R	SMN	STG.R	VAN	< 0.001	−5.05
DCG.L	DMN	STG.R	VAN	< 0.001	−5.15
DCG.R	DMN	STG.R	VAN	< 0.001	−4.88
FFG.R	VIS	STG.R	VAN	< 0.001	−4.63
SMA.L	SMN	TPOsup.L	DMN	< 0.001	−5.47
SMA.R	SMN	TPOsup.L	DMN	< 0.001	−5.25
ACG.L	VAN	TPOsup.L	DMN	< 0.001	−5.58
ACG.R	VAN	TPOsup.L	DMN	< 0.001	−4.74
DCG.L	DMN	TPOsup.L	DMN	< 0.001	−5.19
SMA.R	SMN	TPOsup.R	DMN	< 0.001	−5.03
ACG.L	VAN	TPOsup.R	DMN	< 0.001	−4.85
ACG.R	VAN	TPOsup.R	DMN	< 0.001	−4.54
DCG.L	DMN	TPOsup.R	DMN	< 0.001	−5.04
DCG.R	DMN	TPOsup.R	DMN	< 0.001	−4.77

The table lists all functional connections that showed significantly decreased connectivity in the CLBP group compared to the HCs, surviving a stringent Bonferroni correction for multiple comparisons (overall significance level of *p* < 0.05). For each connection, the two brain regions, their corresponding functional networks based on the Yeo 7-network atlas, the corrected *p*-value, and the *t*-value from the group comparison are provided.

CLBP, Chronic Low Back Pain; FC, Functional Connectivity; ACG, Anterior Cingulate Gyrus; DCG, Dorsal Cingulate Gyrus; FFG, Fusiform Gyrus; HES, Heschl’s Gyrus; INS, Insula; PCL, Paracentral Lobule; PCUN, Precuneus; PHG, Parahippocampal Gyrus; ROL, Rolandic Operculum; SMA, Supplementary Motor Area; STG, Superior Temporal Gyrus; TPOsup, Temporal Pole, Superior Part; L, left; R, right; SMN, Somatomotor Network; VAN, Ventral Attention Network; DMN, Default Mode Network; VIS, Visual Network.

### Group differences in global network metrics

**Figure 2:**
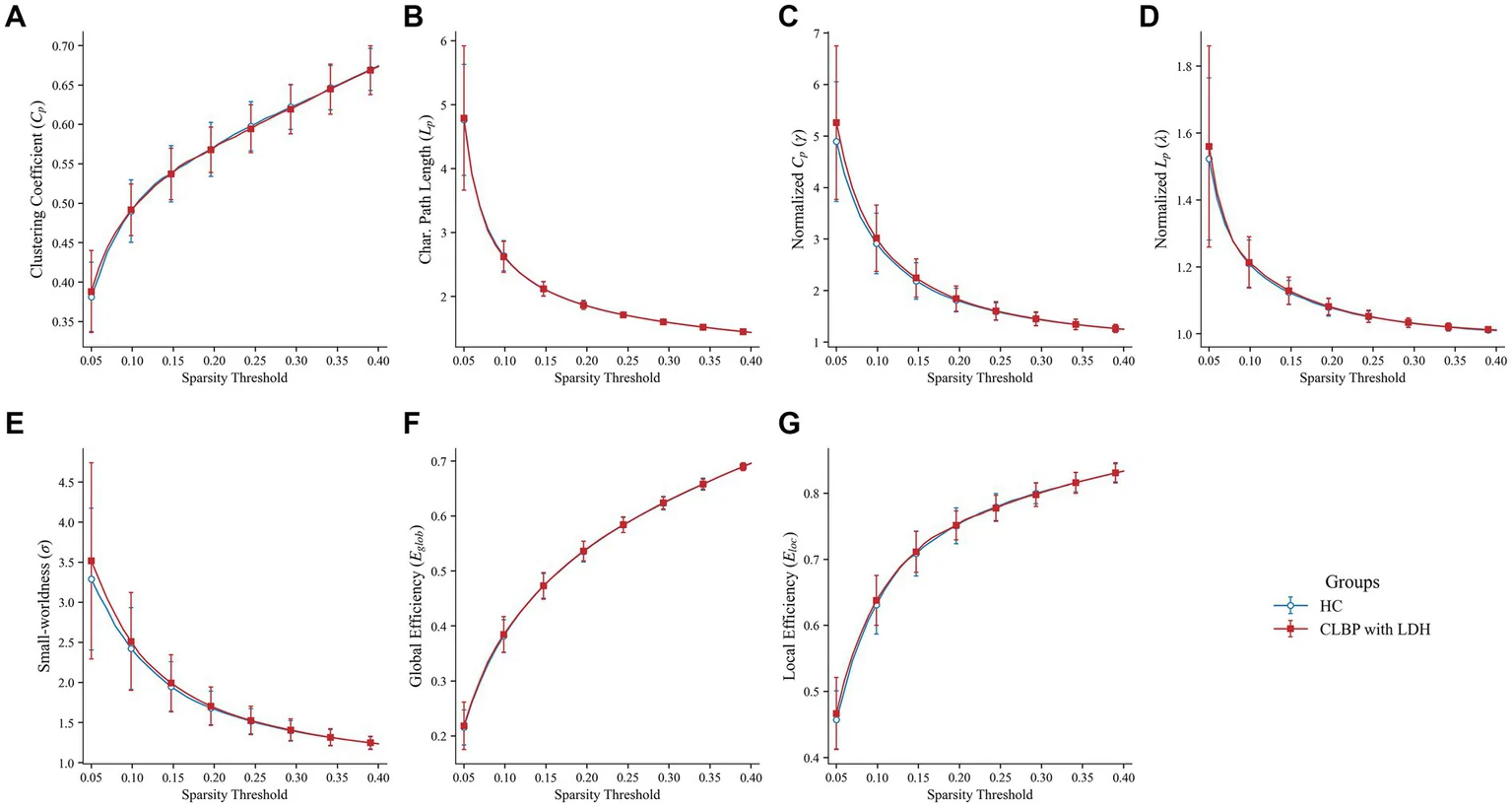
Global topological properties of functional brain networks in the CLBP and HC groups across the sparsity range of 0.05–0.40. The line charts display the changes in global network metrics as a function of network sparsity threshold (0.05–0.40, step = 0.01). **(A)** Clustering coefficient ( $C_{p}$ ); **(B)** Characteristic path length ( $L_{p}$ ); **(C)** Normalized clustering coefficient ( γ ); **(D)** Normalized characteristic path length ( λ ); **(E)** Small-worldness ( σ ); **(F)** Global efficiency ( $E_{\text{glob}}$ ); and **(G)** Local efficiency ( $E_{\mathrm{loc}}$ ). Data are presented as mean ± standard deviation. The red squares represent patients with chronic low back pain (CLBP with LDH), and the blue circles represent healthy controls (HC). Notably, both groups exhibited typical small-world topology ( σ>1 ) across the entire threshold range, but no significant group differences were found in any global metric (all *p* > 0.05, FDR corrected).

The analysis of global topological properties revealed no significant differences between the CLBP and HC groups in any of the calculated metrics, including the clustering coefficient (Cp), characteristic path length (Lp), normalized clustering coefficient (*γ*), normalized characteristic path length (*λ*), small-worldness (*σ*), global efficiency (Eg), and local efficiency (Eloc) (FDR correction for multiple comparisons, all *p* > 0.05). The comparison results of global topology values at different degrees of sparsity are shown in Figure 2.

### Group differences in nodal network metrics

**Figure 3:**
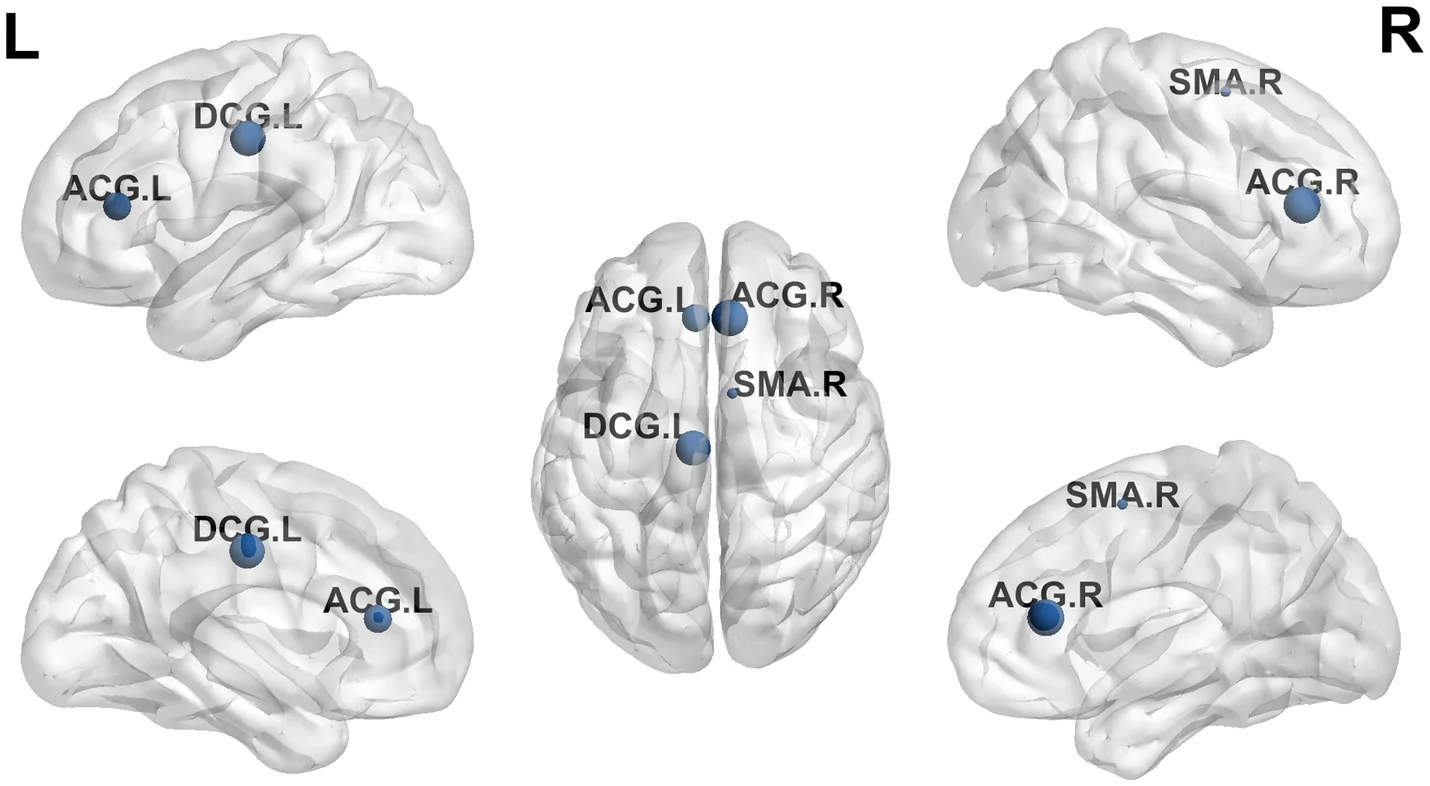
Spatial distribution of brain regions exhibiting altered nodal topological properties in CLBP patients with LDH. All results survived the stringent Bonferroni correction for multiple comparisons (*p* < 0.05, corrected). ACG, Anterior Cingulate Gyrus; DCG, Dorsal Cingulate Gyrus; SMA, Supplementary Motor Area; L, left; R, right.

Compared to HCs, CLBP patients exhibited widespread and significant alterations in nodal topological properties (*p* < 0.001, Bonferroni corrected). Specifically, the bilateral ACG showed significantly reduced betweenness centrality, degree centrality, and nodal efficiency. Furthermore, the DCG. L displayed significantly lower betweenness centrality, and the SMA. R demonstrated reduced nodal efficiency (*p* < 0.001, Bonferroni corrected). These significant alterations in nodal topology are detailed in Table 3 and visualized in Figure 3.

**Table 3:** Group differences in brain network topology.

Brain region	Local metric	*p* value	*t* value
ACG.L	Betweenness Centrality	<0.001	−3.76
	Degree Centrality	<0.001	−4.17
	Nodal Efficiency	<0.001	−4.15
ACG.R	Betweenness Centrality	<0.001	−3.64
	Degree Centrality	<0.001	−4.26
	Nodal Efficiency	<0.001	−4.44
DCG.L	Betweenness Centrality	<0.001	−4.40
SMA.R	Nodal Efficiency	<0.001	−3.61

The table displays local topological metrics that were significantly decreased in the CLBP group compared to the HCs. All reported results survived a stringent Bonferroni correction for multiple comparisons (overall significance *p* < 0.05). Values represent t-statistics from group comparisons. All reported uncorrected *p*-values were less than 0.001. Negative *t*-values indicate a reduction in the metric for the CLBP group.

ACG, Anterior Cingulate Gyrus; DCG, Dorsal Cingulate Gyrus; SMA, Supplementary Motor Area L, left; R, right.

### Correlations with clinical variables

**Figure 4:**
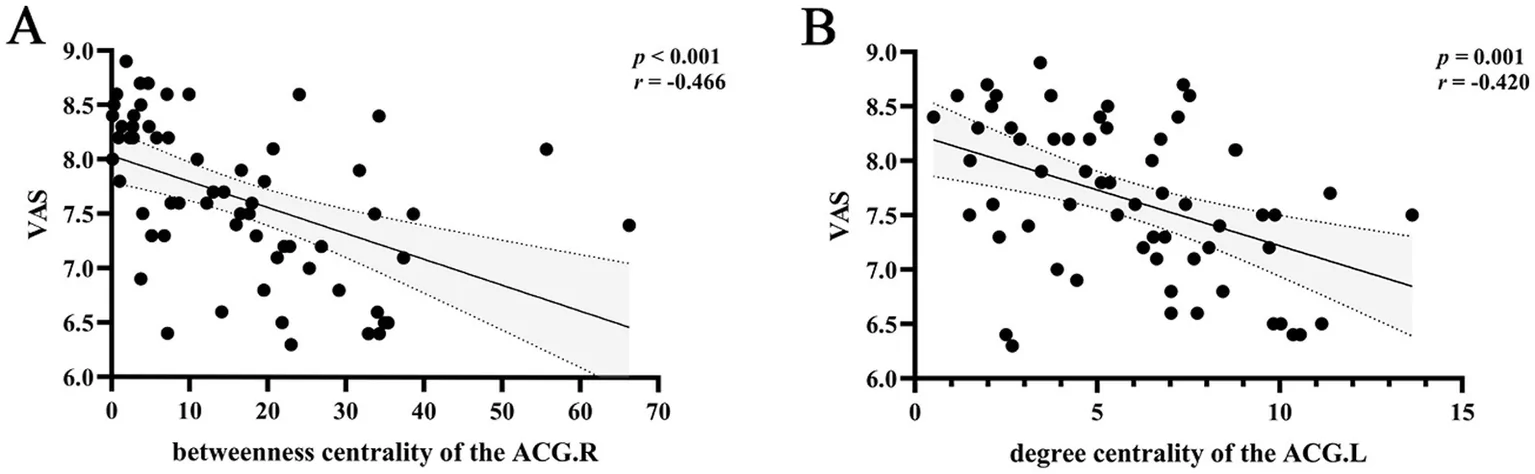
Correlations between altered nodal topological properties and clinical pain intensity. **(A)** Significant negative correlation between the nodal betweenness centrality of the right ACG and VAS scores. **(B)** Significant negative correlation between the nodal degree centrality of the left ACG and VAS scores. Scatter plots display the raw data distribution with linear regression lines (solid) and 95% confidence intervals (shaded). The correlation coefficients ( r ) and p -values displayed were derived from partial correlation analyses, controlling for age, sex, SAS, and SDS scores. All results survived FDR correction (*q* < 0.05). ACG, anterior cingulate gyrus; VAS, Visual Analogue Scale; L, left; R, right.

After rigorously controlling for age, sex, and emotional variables (SAS and SDS scores), correlation analyses revealed robust associations between nodal topology and pain intensity. Specifically, the nodal betweenness centrality of the ACG. R remained significantly negatively correlated with VAS scores (*p* < 0.001, *r* = −0.466). Similarly, the nodal degree centrality of the ACG. L exhibited a significant negative correlation with VAS scores (*p* = 0.001, *r* = −0.420). No other significant correlations were found. These results are visualized in Figure 4.

### Gene set enrichment analysis for altered network topology

**Figure 5:**
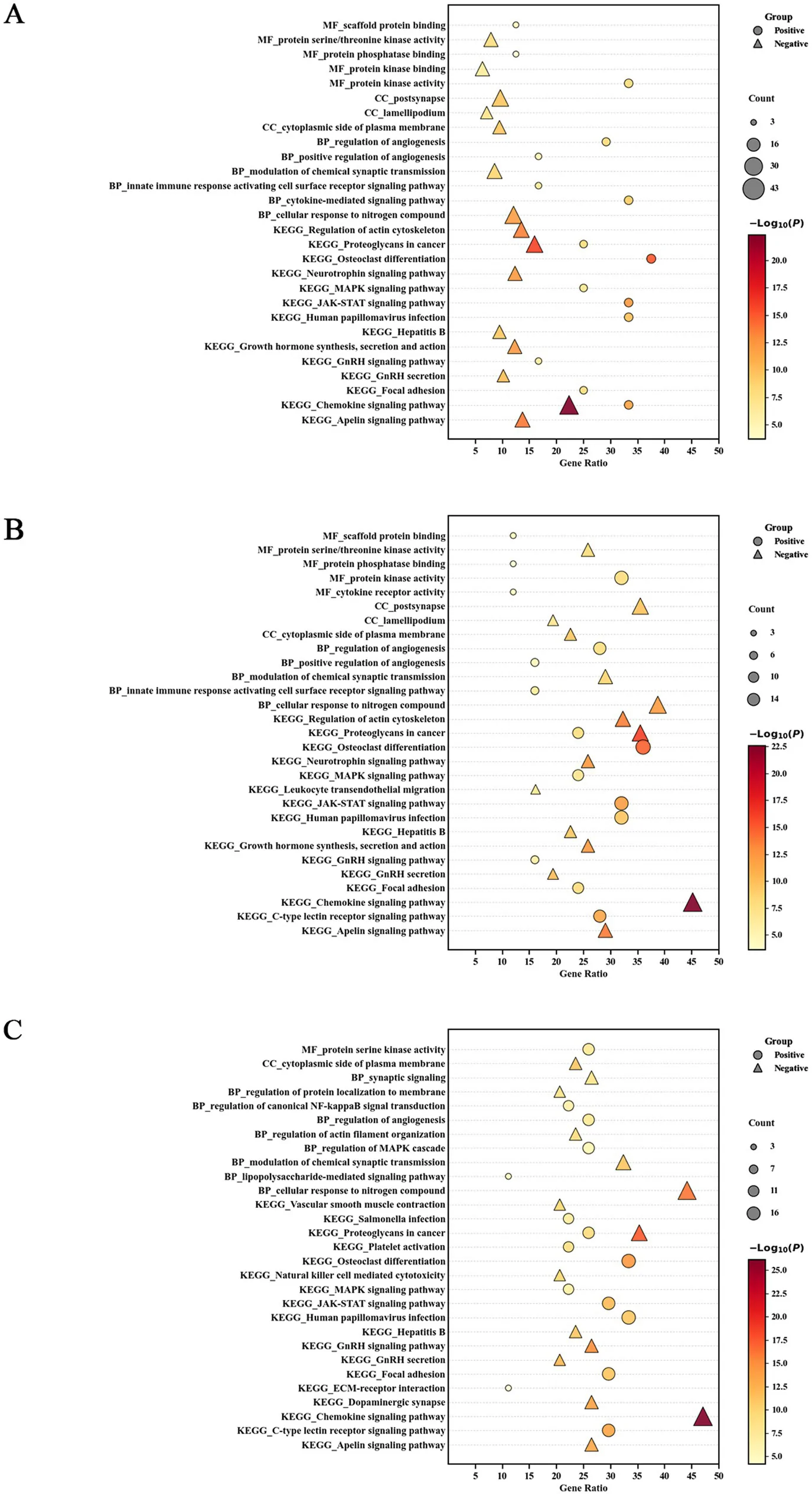
Gene Ontology (GO) and KEGG pathway enrichment analyses of genes associated with aberrant nodal topology via PLS regression. The bubble plots illustrate the significant biological terms and pathways enriched by genes that spatially covaried with **(A)** Nodal Betweenness Centrality, **(B)** Nodal Degree Centrality, and **(C)** Nodal Efficiency. *X*-axis: Represents the Gene Ratio (the ratio of enriched genes to the total number of genes in the pathway). *Y*-axis: Lists the specific GO terms (BP, CC, MF) and KEGG pathways identified via Metascape. Node color: Indicates the statistical significance, scaled as $-\mathrm{Lo}g_{10}(P-\text{value})$ ; redder colors denote higher significance. Node size: Proportional to the number of genes enriched in each term. Node shape: Circles indicate pathways enriched by genes with positive PLS weights (positively associated with the specific nodal metric map), while triangles indicate pathways enriched by genes with negative PLS weights. GO, Gene Ontology; KEGG, Kyoto Encyclopedia of Genes and Genomes; MAPK, Mitogen-Activated Protein Kinase; NF-κB, Nuclear Factor kappa B.

Gene set enrichment analyses revealed that biological processes associated with altered nodal topology in CLBP patients with LDH predominantly converged on two primary themes: neuroinflammation and synaptic plasticity. Specifically, across all three nodal metrics, we observed a consistent enrichment of immune-related pathways, including Chemokine, JAK–STAT, and NF-κB signaling, as well as Leukocyte transendothelial migration. Concurrently, our analyses highlighted a robust enrichment of gene sets critical for synaptic function, evidenced by the over-representation of the “postsynapse” cellular component, molecular functions like “protein kinase activity,” and the MAPK signaling pathway, which is integral to synaptic plasticity (Figure 5).

## Discussion

In this study, we employed a multimodal approach combining rs-fMRI with imaging transcriptomics to systematically investigate functional brain network abnormalities in CLBP secondary to LDH and their potential molecular underpinnings. Our results revealed a multifaceted pattern of network disruption. While global network topology was preserved, significant focal alterations were identified at the nodal level, characterized by markedly reduced degree centrality, nodal efficiency, and betweenness centrality in key regions of the cingulate cortex. These nodal deficits were accompanied by widespread functional hypoconnectivity, primarily between the SMN, DMN, VAN, and VIS networks. Critically, imaging transcriptomics provided a potential biological basis for these macroscopic alterations, linking them to gene expression patterns enriched in immune-inflammatory and synaptic function pathways.

### Widespread decline in FC in CLBP with LDH

Our whole-brain analysis revealed a predominant pattern of widespread functional hypoconnectivity in CLBP patients with LDH compared to HCs, spanning interactions within and between the SMN, VAN, DMN and VIS networks. This finding of large-scale network degradation supports the consensus that chronic pain involves a global disruption of brain communication, reflecting an inefficient network organization dominated by localized dysfunction (Hemington et al., 2015; Baliki et al., 2008; Tu et al., 2019; Huang et al., 2019). Specifically, this decreased connectivity suggests a functional decoupling, where sensory information from the SMN and VIS fails to effectively inform the VAN (Hemington et al., 2015). As the core brain system for appraising the emotional and attentional relevance of these inputs, the compromised integration of this information in the VAN may lead to maladaptive appraisals (Otti et al., 2013; Martucci et al., 2015). This sensory-attentional disconnection likely compromises the critical role of the VAN in detecting salient pain signals and integrating interoceptive bodily sensations with external cues (Hemington et al., 2015). Such impaired integration may directly contribute to clinical phenomena observed in CLBP, including kinesiophobia (where visual cues of movement are decoupled from a sense of bodily safety) and dysfunctional endogenous pain modulation (Hsiao et al., 2017).

In parallel to this sensory-perceptual decoupling, we observed a severe disruption in the brain’s internal cognitive architecture, characterized by impaired VAN-DMN information exchange and significant hypoconnectivity within the DMN itself. The reduced intrinsic connectivity within the DMN likely contributes to the cognitive deficit, while its disconnection from the VAN’s attentional control system allows maladaptive rumination to proceed unchecked (Otti et al., 2013; van der Schaaf et al., 2024; Wortinger et al., 2016). Our finding of resting-state hypoconnectivity contrasts with studies reporting hyperconnectivity in chronic pain (Kolesar et al., 2017; Kim et al., 2019; van Ettinger-Veenstra et al., 2019). This discrepancy highlights the dynamic and heterogeneous nature of chronic pain neurophysiology. While early-stage or neuropathic pain often exhibits hyperconnectivity indicative of central sensitization, our sample had a median pain duration of 3.5 years. Consequently, the observed widespread hypoconnectivity likely represents a state of network decompensation or functional exhaustion following prolonged nociceptive input. This suggests a baseline inefficiency in network organization, which is distinct from the adaptive hyper-responsiveness seen in acute phases or the transient, maladaptive hyper-engagement reported during pain exacerbation (Kim et al., 2019; Lee et al., 2022). The precise DMN-VAN/SMN connectivity patterns are powerfully modulated by individual psychological states, including pain catastrophizing, and may even differ by sex (Kim et al., 2019; Lee et al., 2022; Hidalgo-Lopez et al., 2025). This variability necessitates careful consideration of these factors in future research.

### Regional topological dysfunction in CLBP with LDH

Graph theoretical analysis revealed widespread and significant alterations in nodal topological properties in CLBP patients with LDH compared to HCs. These alterations were not random, but were concentrated in key hubs, including the bilateral ACG, the DCG.L, and the SMA.R. These alterations in network topological properties may help explain the complex nature of the chronic pain experience, which involves sensory and motor impairments.

The cingulate cortex acts as a critical hub intersecting major large-scale brain networks, including the DMN and the SN (Yeo et al., 2011). Within this network framework, it plays a pivotal role in processing the affective-motivational dimensions of pain, rather than its purely sensory components (Vogt, 2005; Xiao et al., 2021). Functionally, the cingulate cortex is subdivided into distinct regions, including the ACG and DCG (Vogt, 2016). The ACG, as a core node of the VAN, is crucial for detecting and evaluating the importance and potential threat of nociceptive signals (Seeley et al., 2007). Building upon this initial evaluation, the DCG acts as a key nexus for cognitive control, integrating these input signals to guide appropriate behavioral and motor responses (Vogt, 2016; Fuchs et al., 2014). This suggests that these specific cingulate subregions are indispensable hubs within the neural architecture of chronic pain. Importantly, the observed functional hub degradation within the cingulate cortex must be interpreted within the context of our patient profile. As presurgical candidates, these patients suffered from severe discogenic radiculopathy, which inherently carries a strong neuropathic pain component. The cingulate cortex plays a pivotal role in the affective and cognitive processing of pain. It is plausible that the continuous nociceptive and neuropathic bombardment from the compromised nerve root induces allostatic overload in the salience network. Consequently, the ‘hub failure’ of the cingulate cortex may reflect functional exhaustion and maladaptive neural plasticity driven by long-term neuropathic distress. Future studies incorporating detailed neuropathic phenotyping (e.g., the DN4 questionnaire) are required to validate this correlation.

Our results align with literature identifying the cingulate cortex as a locus of dysfunction in chronic pain, linking these alterations to clinical severity (Tu et al., 2019; Kano et al., 2020; Ou et al., 2024; Tang et al., 2025; Osborne et al., 2021). However, characterizing this merely as cingulate hypofunction provides an incomplete narrative. This view contrasts with a substantial body of literature, particularly from preclinical models, that reports ACC hyperactivity in chronic pain states (Xiao et al., 2021; Zhuo, 2008; Bliss et al., 2016). This discrepancy likely reflects the complex, multi-layered nature of maladaptive neuroplasticity, rather than a direct contradiction. One compelling explanation is that the hyperactivity may reflect an earlier, active stage of central sensitization common in chronic pain (Zhao et al., 2006; Kang et al., 2012). In contrast, the functional degradation observed in our long-term CLBP sample could signify a later-stage consequence of this sustained excitability as a state of functional exhaustion.

In addition to the aforementioned disruptions in cognitive-affective hubs, our analysis also revealed reduced nodal efficiency in the SMA.R. The SMA is a critical node within the SMN, a system fundamental to motor planning and execution (Qiu et al., 2021). Dysfunction within the SMN is widely reported in chronic pain, including imprecise motor control and a distorted perception of the body (Goossens et al., 2018; Liu et al., 2018). Such dysfunction offers a potential neural mechanism for the motor impairments, altered motor control, and kinesiophobia commonly observed in CLBP patients (Vlaeyen and Linton, 1999; Massé-Alarie et al., 2024).

In summary, our graph theory analysis reveals a significant degradation of nodal topological properties in key brain regions of CLBP patients, specifically within the ACG and SMA. This perspective suggests that the core neuropathology in CLBP is not a simple state of hyper- or hypo-activity in isolated regions, but rather a more complex maladaptive reorganization reflected in the impaired integrative capacity of these crucial hubs.

### Network metrics correlated with clinical pain VAS scores

Our study found that abnormal topological properties of the anterior cingulate network were closely related to patients’ pain experience. The ACG is a critical network hub, playing a core role in evaluating the emotional threat and salience of pain signals and guiding cognitive control (Vogt, 2005; Seeley et al., 2007; Fuchs et al., 2014). Therefore, the observed degradation of this hub function implies an impaired ability of the brain to effectively regulate pain and emotional responses, which directly explains why a poorer hub state is associated with higher perceived pain in patients.

### Microscale mechanisms: neuroinflammation and synaptic plasticity

By integrating imaging transcriptomics, this study provides insights into the potential spatial molecular vulnerabilities associated with macroscale network topology changes in CLBP. Remarkably, the gene expression profiles correlated with all three network properties converged on two core biological processes: sustained neuroinflammatory signaling and maladaptive synaptic plasticity. However, it is crucial to contextualize these findings within the methodological framework of our study. As the gene expression data were derived from healthy donors in the AHBA, our results do not directly reflect altered gene expression within the patients’ brains. Instead, they suggest that the brain regions exhibiting topological vulnerability in CLBP are spatially correlated with a healthy genetic profile enriched for neuroinflammatory and synaptic signaling. This implies that regions with high intrinsic expression of immune-related genes may be more susceptible to functional degradation in the context of chronic pain.

Our analysis revealed that the spatial pattern of altered network topology in CLBP spatially covaried with the normative expression of genes enriched in pathways central to neuroinflammation, including chemokine signaling, the JAK–STAT pathway, leukocyte transendothelial migration, and NF-κB signaling. This spatial colocalization provides strong macro-to-micro support for the hypothesis that brain regions intrinsically rich in these neuroinflammatory profiles may possess a higher spatial vulnerability to pain chronification (Echeverria-Villalobos et al., 2023). This process is characterized by the activation of resident glial cells and the infiltration of peripheral immune cells, which together create a sustained pro-inflammatory microenvironment (Ji et al., 2014; Fiore et al., 2023). The enrichment of these pathways suggests a potential mechanism driven by two complementary sets of pathways. First, the high enrichment of the chemokine signaling pathway points to a widespread inflammatory process involving multiple cell types. Second, the high enrichment of the JAK–STAT pathway reveals how this inflammation is actively maintained and amplified. As the core downstream cascade for cytokine signaling, its enrichment provides the mechanism for a self-sustaining positive feedback loop that perpetuates the inflammatory state (Jacobsen et al., 2020).

This persistent neuroinflammatory environment likely creates a permissive context for maladaptive synaptic plasticity. Inflammatory signaling and peripheral nociceptive input induce long-term potentiation (LTP), a durable enhancement of synaptic efficacy that serves as a hallmark of central sensitization (Bliss et al., 2016; Luo et al., 2014). Pro-inflammatory cytokines act as potent neuromodulators that rapidly enhance excitatory synaptic transmission and promote the establishment of LTP (Ji et al., 2014). This stabilized state of hyperexcitability drives the remodeling of dendritic spines, which in turn strengthens the neural connections relevant to pain processing (Lee et al., 2022; Cui et al., 2022). While this initially drives hyperexcitability, sustained neuroinflammation and excitotoxicity can ultimately lead to synaptic loss and neuronal dysfunction (Rao et al., 2012).

In summary, considering the spatial transcriptomic context, we speculate that brain hubs intrinsically expressing high levels of chemokine-driven neuroinflammatory profiles may be more susceptible to maladaptive synaptic remodeling. Over time, under sustained pathological nociceptive burden, this inherent vulnerability may transition the region from a state of sensitization to one of functional exhaustion, ultimately manifesting as the macroscale degradation of cingulate hub topology observed in our CLBP sample. Crucially, the interpretative boundaries of imaging transcriptomics must be acknowledged. Because the AHBA dataset reflects the baseline gene expression topography of healthy donors, these neuroinflammatory implications represent intrinsic spatial molecular vulnerabilities rather than direct, real-time pathological gene expression. These findings strongly require future empirical validation through peripheral inflammatory biomarker profiling or *in vivo* molecular neuroimaging techniques, such as TSPO-PET for detecting microglial activation (Alshelh et al., 2022; Loggia, 2024; De Picker et al., 2023).

### Limitations and future directions

There are several limitations in this research. First, our cross-sectional design precludes causal inference. Whether the observed brain alterations are a predisposing factor for or a consequence of chronic pain remains unclear. Longitudinal studies tracking patients from acute to chronic stages are essential to resolve this. Second, the imaging transcriptomics approach is indirect, correlating patient fMRI data with a healthy donor brain atlas (AHBA). This method assumes that spatial gene expression topography is stable; therefore, our findings should be strictly interpreted as reflecting a spatial transcriptional vulnerability rather than confirming real-time altered gene expression within the patients’ brains. The robustness of these findings is further constrained by the AHBA’s small sample size and its primary restriction to the left hemisphere. Third, regarding medication effects, although a rigorous 24-h washout period was implemented to eliminate acute pharmacological influences on the BOLD signal, we cannot entirely rule out the potential long-term neuroplastic effects of chronic analgesic use on brain network topology. Due to the reliance on retrospective medical records and patient recall for historical medication tracking, granular data on the exact lifetime dosage and precise duration of prior substance use were heterogeneous and unavailable, preventing further subgroup sensitivity analyses. Future longitudinal studies involving strictly drug-naïve groups would be valuable to disentangle these factors. Fourth, we utilized the AAL-90 atlas to define network nodes. While the AAL atlas ensures direct comparability with a wealth of existing chronic pain literature and provides robust coverage of essential subcortical pain matrices, its macro-anatomical boundaries inevitably aggregate functionally distinct sub-regions of the cingulate cortex (anterior, middle, and posterior). The fact that profound ‘hub degradation’ was detectable even at this macro-scale suggests a sweeping topological disruption. Nevertheless, future studies utilizing higher-granularity functional templates (such as the Schaefer or Power atlases) are highly encouraged to map the micro-functional topography and validate these findings. Finally, while our focus on a homogenous presurgical patient sample (LDH) enhances internal validity, it may limit the generalizability of our findings to the broader, highly heterogeneous CLBP population. Future research incorporating diverse CLBP subtypes is warranted.

## Conclusion

In conclusion, this study elucidates the central neuropathology of CLBP secondary to LDH, identifying significant disruptions at both the topological and connectivity levels. Our findings highlight a specific central mechanism characterized by degraded cingulate hub properties that correlate with clinical severity, alongside widespread functional hypoconnectivity between key large-scale networks. Critically, by integrating imaging transcriptomics, we uncover potential spatial transcriptional vulnerabilities associated with this central network reorganization, linking it to a biological cascade of sustained neuroinflammation and maladaptive synaptic plasticity. This comprehensive analysis, which connects macroscale network dysfunction to underlying molecular mechanisms, underscores the critical role of CNS maladaptation in LDH-induced pain, promising to inform future mechanistic research and novel therapeutic strategies.

## Data Availability

The raw data supporting the conclusions of this article will be made available by the authors, without undue reservation.

## Footnotes

1
https://metascape.org
