# Supplementary material for: Transcriptional signatures of aberrant brain functional network topology in chronic low back pain with lumbar disc herniation
Source: Front Mol Neurosci. 2026 Aug 12;19:1790428. doi: 10.3389/fnmol.2026.1790428 (PMC13506785; doi:10.3389/fnmol.2026.1790428)
Supplement: Supplementary file 1 [file Table_1.docx]

Table S1. Clinical characteristics of patients with LDH-related CLBP.

| **Characteristics** | **Number of patients (n = 62)** | **Percentage (%)** |
| --- | --- | --- |
| **Affected spinal levels** |  |  |
| Single level (e.g., L4-L5, L5-S1) | 28 | 45.2% |
| Multiple levels (e.g., L3-S1, L4-S1) | 34 | 54.8% |
| **Laterality of herniation** |  |  |
| Central / No obvious laterality | 44 | 71.0% |
| Left-sided | 12 | 19.4% |
| Right-sided | 5 | 8.1% |
| Unknown | 1 | 1.6% |
| **Nerve root compression** |  |  |
| Absent | 50 | 80.6% |
| Present (e.g., lateral recess stenosis) | 12 | 19.4% |
| **Pain distribution** |  |  |
| Localized to lower back | 57 | 91.9% |
| Radiating to lower extremities | 5 | 8.1% |
| **Prior treatments** (Surgical/Interventional) |  |  |
| *Any surgical intervention* | 19 | 30.6% |
| - Discectomy / Nucleotomy | 3 | 4.8% |
| - Internal fixation / Fusion | 6 | 9.7% |
| - Unspecified post-operative state | 10 | 16.1% |
| **Prior treatments (Analgesics)** |  |  |
| - NSAIDs | 45 | 72.6% |
| - Gabapentinoids | 10 | 16.1% |
| - Over-the-counter (OTC) analgesics | 7 | 11.3% |

**Abbreviations:** CLBP, chronic low back pain; LDH, lumbar disc herniation; NSAIDs, non-steroidal anti-inflammatory drugs.
